# AGR3 Regulates Airway Epithelial Junctions in Patients with Frequent Exacerbations of COPD

**DOI:** 10.3389/fphar.2021.669403

**Published:** 2021-06-11

**Authors:** Rui Ye, Cuihong Wang, Pengbo Sun, Shuang Bai, Li Zhao

**Affiliations:** Department of Pulmonary and Critical Care Medicine, Shengjing Hospital of China Medical University, Shenyang, China

**Keywords:** COPD, AGR3, frequent exacerbator, human lung tissue, airway epithelial barrier

## Abstract

**Background:** The mechanisms underlying differences in the susceptibility to chronic obstructive pulmonary disease (COPD) exacerbations between patients are not well understood. Recent studies have shown that the patients with frequent COPD exacerbations is related to specific protein expression in lung tissue. Anterior gradient 3 (AGR3) is expressed in airway epithelial cells in the lung and proteomic analysis revealed that its expression is decreased in patients with frequent COPD exacerbations. Moreover, the loss of epithelial integrity might facilitate trans-epithelial permeability of pathogens in such patients. This study was performed to determine that AGR3 protein play a role in COPD frequency exacerbators.

**Methods:** Human lung tissues were collected from current-smoking patients (Control; n = 15) as well as patients with infrequent COPD exacerbations (IFCOPD; n = 18) and frequent COPD exacerbations (FCOPD; n = 8). While AGR3 protein expression was measured by immunohistochemistry and western blotting, *AGR* mRNA expression was determined by real time quantitative polymerase chain reaction (RT-qPCR). Furthermore, adherent junctions (AJs) and tight junctions (TJs) protein expression in human lung tissues were measured by immunohistochemistry. The effects of cigarette smoke extract (CSE) on AJ and TJ protein and mRNA expression in BEAS-2B cells were assessed by western blotting and RT-qPCR. In addition, the effect of AGR3 overexpression and knockdown on AJ and TJ protein expression was determined.

**Results:** AGR3 was mainly expressed in the airway epithelium and AGR3-positive products were localized in the cytoplasm. Western blotting and RT-qPCR results showed that AGR3 protein (*p* = 0.009) and mRNA (*p* = 0.04) expression in the FCOPD group was significantly lower than that in the IFCOPD group. Moreover, E-cadherin, occludin, and zonula occludens-1 (ZO-1) expression was lower in the FCOPD group than in the IFCOPD group. The protein and mRNA expression of E-cadherin, occludin, and ZO-1 was decreased within 24 h post-CSE exposure. AGR3 overexpression rescued CSE-induced downregulation of E-cadherin, occludin, and ZO-1.

**Conclusion:** Difference in AGR3 expression in the lung tissue might be correlated with increased susceptibility to COPD exacerbation. AGR3 can prevent CSE-induced downregulation of E-cadherin, occludin, and ZO-1 in airway epithelial cells. Loss of AGR3 might promote viral and bacterial infection and induce immune inflammation to increase COPD exacerbation.

## Introduction

Chronic obstructive pulmonary disease (COPD) is a worldwide public health problem that accounts for 5% of all deaths each year and is associated with increasing morbidity and mortality (Wang et al., 2018). Among various COPD phenotypes, the frequent-exacerbation phenotype has the most marked impact on accelerating the decline of lung function, reducing physical activity, decreasing quality of life, and increasing mortality (Donaldson et al., 2002). The frequent-exacerbation phenotype of COPD was first described by Hurst et al., in 2010 and its clinical importance was demonstrated (Hurst et al., 2010; From the Global Strategy for the Diagnosis, Management and Prevention of COPD, Global Initiative for Chronic Obstructive Lung Disease (GOLD), 2018). So far it is no primary outcome that reduces the frequency and severity of COPD exacerbations for almost any development of new drugs in COPD, especially for target therapies. Unfortunately, two kinds of monoclonal antibodies that bind to receptors related to eosinophil reduction were found to be ineffective in their clinical trials in COPD patients, including frequent exacerbation phenotype (Pavord et al., 2017; Criner et al., 2019). The underlying mechanisms and predisposing factors of the frequent-exacerbation COPD phenotype are still unclear. A previous study on the proteomics of lung tissues in patients with frequent COPD exacerbations identified 23 differentially expressed proteins (Sun et al., 2019), including anterior gradient 3 (AGR3).

The AGR protein family consists of three subfamilies: AG1, AGR2, and AGR3. The AGR family proteins share high sequence homology to the non-secreted protein disulphide isomerase (PDI) family proteins (Ivanova et al., 2013). The PDI family proteins harbor core thioredoxin folds (CxxC or CxxS motifs), which can promote protein folding via modulation of disulphide bond formation and calcium homeostasis in the endoplasmic reticulum (Persson et al., 2005). AGR3, also known as HAG-3, AG3, or BCMP11, was first identified in breast cancer cell membrane fractions using a proteomics screen (Adam et al., 2003). AGR3 is a highly related homologue of pro-oncogenic AGR2, and both genes are located on chromosome 7p21 (Petek et al., 2000). The effects of AGR3 on ciliary beat frequency in airway epithelial cells were demonstrated using a knockout mouse model (Bonser et al., 2015). However, limited information is available about AGR3 function in the respiratory system. AGR3 is involved in mucociliary system which plays an important role in defense to foreign pathogens. Epithelial tight junctions (TJs) and adherens junctions (AJs) also plays an important role in host defense against foreign pathogens (Nawijn et al., 2011). The disruption of these structures might increase the susceptibility of patients with COPD to viral and bacterial infection. Most patients with COPD exacerbations have viral and bacterial infection (Hiemstra et al., 2015). To our knowledge, the effect of AGR3 on airway epithelial barriers in patients with frequent COPD exacerbations has not been studied. The present study was conducted to identify potential therapeutic targets of patients with frequent COPD exacerbations. Furthermore, AGR3 expression was measured in the lung tissues of patients with frequent and infrequent COPD exacerbations and the role of AGR3 on epithelial junctions was determined both in lung tissues and vitro.

## Materials and Methods

### Lung Tissue Sample Collection

The study was approved by the Ethics Review Committee for Human Studies at Shengjing Hospital of China Medical University, and informed written consent was obtained from each patient (2016PS342K). The inclusion criteria for patients with COPD were as follows: 1) 40–80 years of age; 2) history of 10 or more pack-years of smoking; 3) ratio of forced expiratory volume in 1 s (FEV1) to forced vital capacity (FVC) (FEV1/FVC ratio) of 70% or less after bronchodilator use (Wang et al., 2019); and 4) stable clinical pulmonary condition of COPD, with no acute exacerbations for at least 4 weeks prior to enrollment. The severity of airway limitation was graded based on FEV1post%, as described in the GOLD guidelines (Wang et al., 2018). Patients with COPD were assigned to two groups based on their medical history: infrequent exacerbations (IFCOPD) and frequent exacerbations (FCOPD). Patients in the FCOPD group must have experienced at least two or more exacerbations per year that required treatment with additional antibiotics or a systemic steroid, or hospitalization (Wang et al., 2018). Each exacerbation must have occurred at least 4 weeks after the end of treatment for the previous exacerbation or 6 weeks after its onset (Spencer and Jones, 2003; Soler-Cataluña and Rodriguez-Roisin, 2010). These criteria were included to distinguish a new exacerbation from a “treatment failure” (Soler-Cataluña and Rodriguez-Roisin, 2010). In addition, subjects with normal lung function and current-smoking history >10 pack-years were enrolled as the control group. Patients with other respiratory diseases such as asthma, pulmonary fibrosis, and hilar lung cancer, or other chronic diseases such as rheumatoid arthritis and chronic colitis were excluded from the study. Two professional pulmonologists assembled a detailed clinical history for each patient and conducted comprehensive lung function tests using standard spirometric techniques. Lung tissue specimens were retrieved from Biobank, Shengjing Hospital of China Medical University. All biopsy tissues were obtained from patients who underwent pulmonary lobectomy. These resected tissue samples included non-diseased regions near lung nodules and tumors, bullectomy (at least 5 cm from the tumor edge) and appeared to be normal when inspected as previously described (Bi et al., 2015). The location and amount (approximately 1 cm^3^) of each sample were standardized and paired according to tumor type and grade (Kelsen et al., 2008). The resected samples were immediately frozen in liquid nitrogen and stored at −80°C until use.

### Immunofluorescence and Immunohistochemistry

Lung tissue blocks were fixed in 10% neutral buffered formalin for 24 h and embedded in paraffin wax. Immunohistochemical analysis was performed on 2.5-μm-thick sections cut from formalin-fixed, paraffin-embedded archival tissue blocks. These sections were mounted on slides, deparaffinized in xylene, and rehydrated in phosphate-buffered saline (PBS) through a graded ethanol series. Endogenous peroxidase activity was quenched in 3% H_2_O_2_ in PBS for 15 min. Antigen retrieval was performed in citrate buffer, pH 6 at 100°C for 7.5 min. For AGR3 immunodetection, the sections were incubated overnight at 4°C with rabbit monoclonal anti-AGR3 (1:200, Proteintech, Chicago, IL, United States), anti-E-cadherin (1:1,000, Proteintech), anti-ZO-1 (1:400, Proteintech), and anti-occludin (1:500, Proteintech). A streptavidin–biotin peroxidase detection system was used according to the manufacturer’s protocol (Ultrasensitive™ S-P Kit, Maxim Biotechnologies, Fuzhou, China) for immunohistochemical studies. In addition, each section was stained using the DAB color reagent kit (ZSGB-BIO, Beijing, China).

For immunofluorescence, primary antibodies were rabbit monoclonal anti-AGR3 (1:200, Proteintech), monoclonal anti-cytokeratin (1:100, Sigma-Aldrich, Darmstadt, Germany), rabbit anti-E-cadherin (1:100, Proteintech), anti-ZO-1 (1:100, Proteintech), and anti-occludin (1:100, Proteintech) and Alexa Fluor-conjugated secondary antibodies were goat anti-rabbit (488 nm) (1:100, Proteintech), goat anti-mouse (594 nm) (1:100, Proteintech) and donkey anti-rabbit (594 nm) (1:100, Sigma-Aldrich). Immunofluorescence staining was visualized under Olympus FV1000 confocal microscope with a 60× oil-immersion objective. Images were acquired and analyzed by NIS-Elements F 3.0 and NIS- Elements Br 3.0.

### Western Blot

Total protein was extracted from frozen lung tissues using RIPA and phenylmethylsulfonyl fluoride (PMSF) (99:1) (Beyotime, Shanghai, China). Protein concentrations were determined using the BCA kit (Beyotime). Protein extracts were subjected to SDS-PAGE and visualized with SuperSignal West Pico Chemiluminescent substrate (Thermo Fisher, MA, United States). The following primary antibodies were purchased from Proteintech and used for western blotting: anti-GAPDH (1:5,000), anti-AGR3 (1:500), anti-E-cadherin (1:8,000), anti-occludin (1:5,000), and anti-ZO-1 (1:5,000).

### Real Time Polymerase Chain Reaction

The primers used for real time polymerase chain reaction (RT-PCR) are shown in Table 1. Total RNA was extracted from lung tissues using TRIzol reagent (Takara, Ohtsu, Shiga, Japan), according to the manufacturer’s instructions. The transcribed cDNA concentration was adjusted to 120 ng/ml with RNase-free water before PCR analysis. PCR was performed using the SYBR Green PCR Kit (Takara) with a Roche 480 Real-Time PCR System (Roche, Basel, Switzerland). The PCR cycling parameters were as follows: pre-incubation for 2 min at 42°C and 40 cycles of denaturation at 95°C for 5 s. RT-PCR was performed in triplicate for both endogenous controls and each target gene. Relative gene expression was calculated using the 2^−△△ct^ method. Melting curve analysis and 2% agarose gel electrophoresis were performed to confirm the PCR product.

**TABLE 1:** RT-PCR primers.

Gene	Sequence	Length (nt)
*GAPDH-F*	5′-CCT​GGT​ATG​ACA​ACG​AAT​TTG-3′	131
*GAPDH-R*	5′-CAG​TGA​GGG​TCT​CTC​TCT​TCC-3′	
*AGR3-F*	5′-CAT​CAC​CTG​GAG​GAT​TGT​CAA​TAC-3′	91
*AGR3-R*	5′-TGA​ACT​TAT​TCT​GAG​CCA​TTT​CTT​GT-3′	
*Occludin-F*	5′-TGC​ATG​TTC​GAC​CAA​TGC-3′	235
*Occludin-R*	5′-AAG​CCA​CTT​CCT​CCA​TAA​GG-3′	
*ZO-1-F*	5′-AAG​ATG​TCC​GCC​AGA​GCT​GC-3′	80
*ZO-1-R*	5′-AGC​GTC​ACT​GTA​TGT​TGT​TCC​C-3′	
*E-cadherin-F*	5′-AGG​GGT​TAA​GCA​CAA​CAG​CA-3′	161
*E-cadherin-R*	5′-GGT​ATT​GGG​GGC​ATC​AGC​AT-3′	

### Cell Culture

BEAS-2B (CRL-9609) cell line was obtained from the Cell Bank of the Chinese Academy of Sciences (Shanghai, China) and cultured in high-glucose Dulbecco’s Modified Eagle’s Medium (H-DMEM) supplemented with 10% foetal bovine serum, 100 U/ml penicillin, and 100 mg/ml streptomycin (growth medium) at 37°C in a 5% CO_2_, 95% air atmosphere. Unless otherwise stated, cells were grown to 70–80% confluency prior to treatment.

### Plasmid Construction and Cell Transfection

For lentiviral AGR3 overexpression in BEAS-2B cells, full-length *AGR3* was cloned into the pHBLV-CMV-MCS-3FLAG-EF1-ZsGreen-T2A-PURO vector. Lentivirus packaging, cellular transfection, and selection of puromycin-resistant cells were performed according to the instruction manual provided by Hanbio Biotechnology (Shanghai, China). shAGR3 and shNC were purchased form GenePharma (Suzhou, China). Cells were transfected with Lipofectamine 3,000 (Invitrogen), following the product manual. The nucleotide sequences of all constructed plasmids were confirmed by DNA sequencing.

### Cigarette Smoke Extract Preparation

Cigarette smoke extract (CSE) was prepared by bubbling smoke from two filtered cigarettes (Hongmei cigarettes, tobacco type, tar: 10 mg, nicotine content: 0.8 mg, and carbon monoxide fumes: 12 mg; Hongta Tobacco Industry LLC, Yunnan, China) in a 50-ml centrifuge tube containing 20 ml H-DMEM by vacuum extraction. The extracts were sterilized by passing through a 0.22-μm filter and considered full concentrations of CSE (Kelsen et al., 2008).

### Statistical Analysis

Statistical analyses were performed using SPSS 22.0 software (Chicago, IL, United States) and GraphPad Prism 7 (La Jolla, CA, United States). Data are expressed as the mean ± SD. ANOVA was used to compare differences between groups. *p* < 0.05 was considered statistically significant.

## Results

### Patient Information

Lung tissues from 41 patients (15 controls, 18 IFCOPD, and 8 FCOPD) were collected from September 2016 to September 2018. There were no significant differences in the age or gender between the three groups. In addition, there were no significant differences in FEV1 (%predict), FVC (%predict), and FEV1/FVC between the IFCOPD and FCOPD groups (Table 2).

**TABLE 2:** Demographic characteristics of patients.

	Control	IFCOPD	FCOPD	*p*
	(n=15)	(n=18)	(n=8)	
Age	57.87 ± 6.97	59.00±6.94	65.00±6.41	0.061
Gender (M/F)	11/4	15/3	7/1	
Packs/year	26.67 ± 15.99	30.61 ± 15.67	37.50 ± 11.65	0.275
FEV1 (%predict)	100.49 ± 18.27	71.80 ± 14.75	57.92 ± 14.95	0.051^a^
FVC (%predict)	101.57 ± 12.68	91.91 ± 15.48	80.06 ± 15.44	0.062^a^
FEV1/FVC	80.77 ± 10.26	62.48 ± 6.65	56.86 ± 5.97	0.113^a^

All values are represented as mean ± SD.

a *p* > 0.05 in FEV1 (%predict), FVC(%predict), and FEV1/FVC between IFCOPD and FCOPD groups.

### AGR3 Is Downregulated in the Airway Epithelial Cells of Patients with Frequent COPD Exacerbation

**FIGURE 1:**
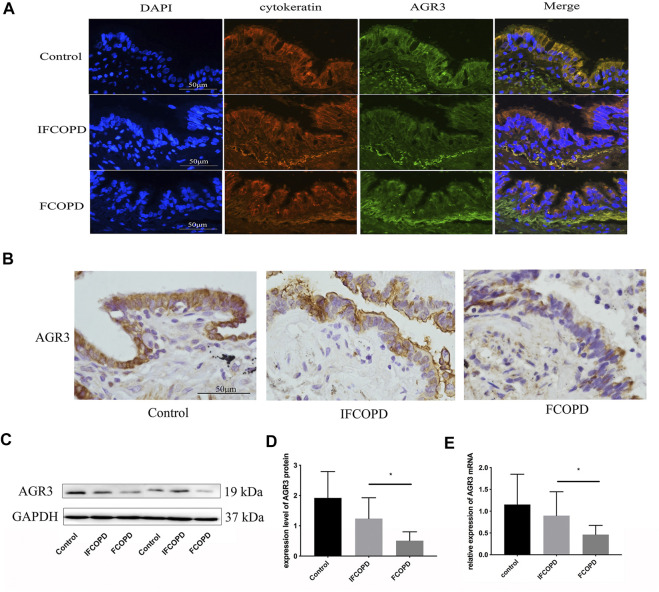
AGR3 expression in airway epithelial cells of frequent COPD exacerbators. The control group included subjects with normal lung function and a current smoking history of >10 pack years. **(A)** Immunofluorescence staining for AGR3 (green) and cytokeratin (red) in human airway epithelial cells **(B)** Immunohistochemical staining for AGR3 in human airway epithelial cells of Control (n = 15), IFCOPD (n = 18), and FCOPD (n = 8) groups. The control group included subjects with normal lung function and a current smoking history of >10 pack years. Cells were stained with anti-AGR3 antibody. AGR3 expression in the airway epithelium of patients with COPD and control subjects was determined by IHC (400×). **(C,D)** Western blot analysis of AGR3 expression in FCOPD, IFCOPD, and control groups. ∗*p* < 0.05. **(E)** qPCR analysis of *AGR3* mRNA expression in lung tissues of FCOPD, IFCOPD, and control groups. ∗*p* < 0.05.

Histological analysis showed that AGR3 is mainly expressed in the airway epithelial cells of the lung tissue and AGR3-positive products are localized in the cytoplasm (Figure 1A). AGR3 protein expression in the IFCOPD and FCOPD groups was significantly lower than that in the control group (Figures 1A,B, *p* = 0.009). Moreover, *AGR3* mRNA expression in the FCOPD group was lower than that in the IFCOPD group (Figure 1C, *p* = 0.04).

### Epithelial Junction Proteins are Downregulated in Patients with Frequent COPD Exacerbation

**FIGURE 2:**
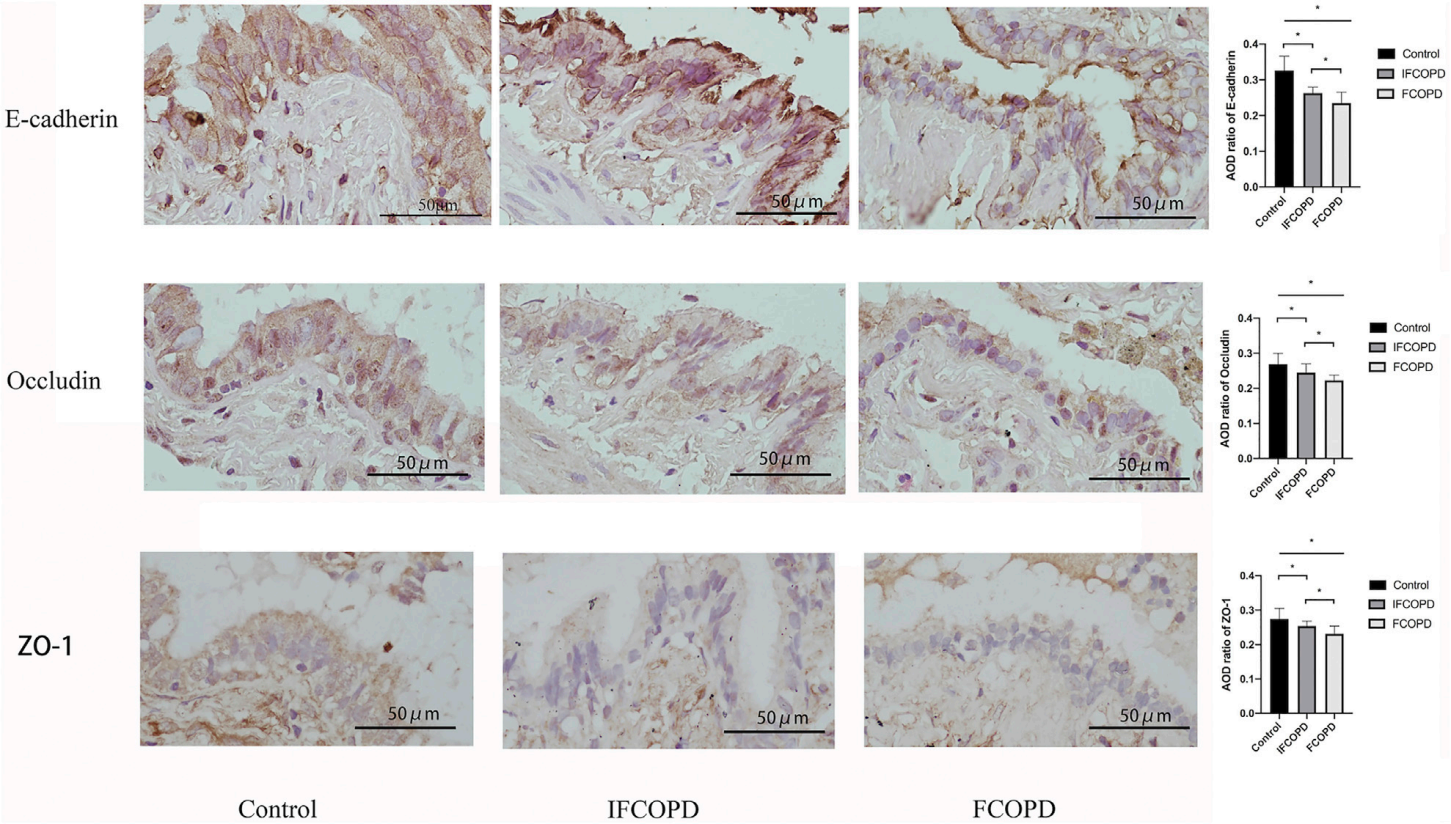
Immunohistochemical analysis of E-cadherin, occludin, and ZO-1 expression in the airway epithelium cells of Control (n = 15), IFCOPD (n = 18), and FCOPD (n = 8) groups. The control group included subjects with normal lung function and a current smoking history of >10 pack years. Cells were stained with anti-E-cadherin, anti-occludin, and anti-ZO-1 antibody. All subjects were determined by IHC (400×). Data are displayed as mean ± SD, *p < 0.05 by one-way ANOVA. Results represent significant differences in the expression of E-cadherin, occludin, and ZO-1 in airway epithelium cells, compared to the Control group.

Epithelial junction proteins in the bronchial epithelium consist of apical TJ proteins such as occludins, zonula occludins (ZO), and claudin family and junctional adhesion molecule, and AJ proteins such as E-cadherin (Heijink et al., 2014). The expression of E-cadherin, occludin, and ZO-1 was assessed by immunohistochemistry in the FCOPD and IFCOPD groups. Compared to Control group, the expression of E-cadherin in airway epithelial cells was decreased in COPD groups (*p* = 0.004) and markedly lower in IFCOPD group (*p* = 0.006). As same as the expression of E-cadherin, the expression of occludin, and ZO-1 was respectively decreased in COPD groups (*p* = 0.001and *p* = 0.001), and markedly lower in IFCOPD group (*p* = 0.03 and *p* = 0.004) (Figure 2).

### CSE Downregulates TJ and AJ Proteins in Airway Epithelial Cells

**FIGURE 3:**
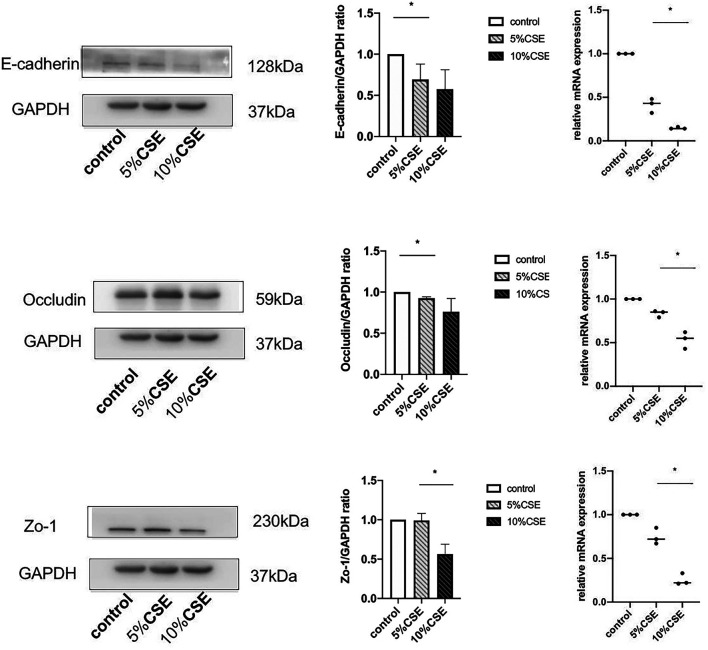
Western blotting and real-time quantitative reverse-transcriptase PCR results showing TJ and AJ protein expression in BEAS-2B cells in the presence or absence of 5 and 10% CSE for 24 h. Band intensity was quantitated using densitometry. All results are representative of at least three independent experiments. Data are shown as the mean ± SD. *p < 0.05 by one-way ANOVA.

The effects of CSE exposure on the expression of TJ proteins (occludin and ZO-1) and an AJ protein (E-cadherin) were determined. BEAS-2B cells were treated with 5 and 10% CSE for 24 h. The gene and protein expression of occludin, ZO-1, and E-cadherin were analyzed by qPCR and western blotting, respectively. The treatment of cells with 10% CSE significantly decreased gene and protein expression of occludin, ZO-1, and E-cadherin (Figure 3).

### AGR3 Rescue CSE-Induced Downregulation of AJs and TJs in Airway Epithelial Cells

**FIGURE 4:**
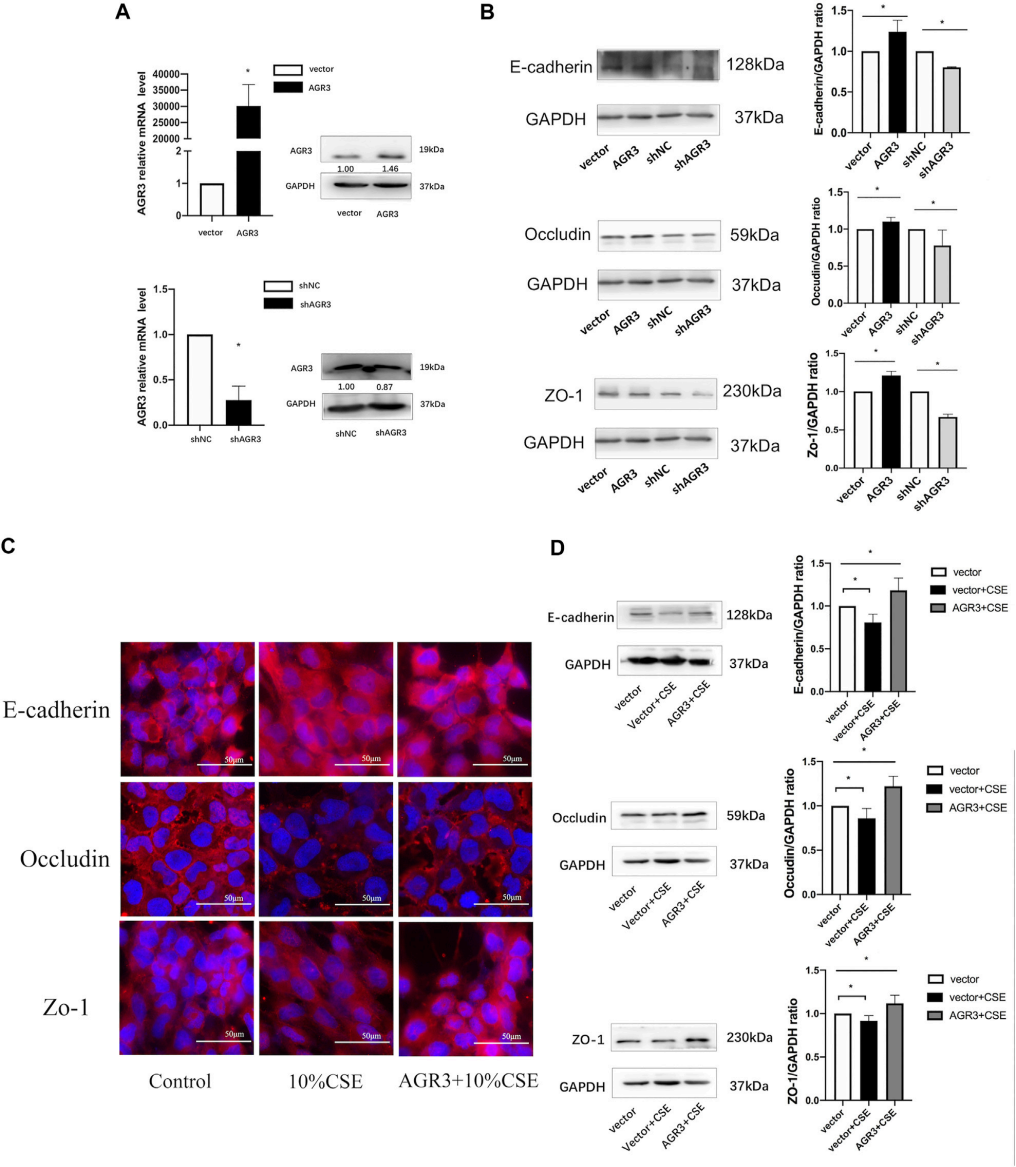
Effects of AGR3 on E-cadherin, Occludin, and ZO-1 expression in BEAS-2B cells. **(A)** AGR3 overexpressed and silenced in BEAS-2B cells. **(B)** Protein expression of occludin, ZO-1, and E-cadherin in AGR3 overexpressed and silenced BEAS-2B cells. **(C)** Immunofluorescence staining for E-cadherin, Occludin, and ZO-1 in Control cells or AGR3 overexpressed cells with 10% CSE exposed for 24 h. **(D)** Western blot analysis for E-cadherin, Occludin, and ZO-1 in Control cells or AGR3 overexpressed cells with 10% CSE exposed for 24 h. Band intensity was quantitated using densitometry. All results are representative of at least three independent experiments. Data are shown as the mean ± SD. *p < 0.05 by one-way ANOVA.

The ability of AGR3 to rescue CSE-induced downregulation of TJs and AJs in bronchial epithelial cells was assessed. At first, *AGR3* was overexpressed or silenced in BEAS-2B cells. Western blot analysis showed that the expression of occludin, ZO-1, and E-cadherin was significantly increased in AGR3-overexpressed BEAS-2B cells and decreased in AGR3-silenced BEAS-2B cells. Next, the ability of AGR3 to rescue CSE-induced downregulation of TJ and AJ proteins was determined. Western blot analysis showed increased expression of occludin, ZO-1, and E-cadherin in CSE-exposed AGR3-overexpressing cells (Figure 4).

## Discussion

COPD is characterized by airway inflammation with periods of symptom exacerbation that leads to irreversible airflow limitation, lung damage, and mucus hypersecretion. The treatment strategies for patients with COPD remain a challenge due the wide range of COPD phenotypes and their unpredictable clinical course (Wang et al., 2018). The frequency of COPD exacerbation can determine COPD phenotype. Patients with more than two exacerbations per year have been defined as having frequent COPD exacerbations. These individuals have high levels of inflammatory cytokines such as interleukin-6 in sputum (Bhowmik et al., 2000), and show a high risk of adverse health outcomes. In our previous study on patients with frequent COPD exacerbations, proteomics analysis showed low expression of several proteins in lung tissues (Sun et al., 2019). Moreover, AGR3 was significantly decreased and previous study (Bonser et al., 2015) showed AGR3 expressed in the respiratory airway epithelium.

In this study, we first measured AGR3 expression in the lung tissue of patients with frequent COPD exacerbations. Histochemical analysis revealed that AGR3 proteins are predominantly expressed in airway epithelial cells. In addition, AGR3 protein and mRNA expression in lung tissues of patients with frequent COPD exacerbations was significantly lower than that of patients with infrequent COPD exacerbations. Thus, AGR3 protein and gene expression is downregulated in patients with frequent COPD exacerbations.

AGR3 belongs to the PDI family and is a highly related homologue of pro-oncogenic AGR2. Unlike AGR2, the functional role of AGR3 remains unclear. A previous study has shown that AGR3 is expressed in ciliated cells and is required for mucociliary clearance and calcium-modulated ciliary beat frequency (Bonser et al., 2015). Airway epithelial barriers are required for innate immunity as the first line of defense against microbial invasion in the lung. Other studies have shown that COPD at a high risk of exacerbations is associated with excessive microbiota colonization in the lower respiratory track (Miravitlles et al., 1999; Yawn et al., 2013). Thus, we speculated that the downregulation of AGR3 might predict the reduction of airway microbiota clearance or innate immune system, and then inducing lung microbiota easy to be inhaled into low respiratory track and colonized. These event increases the risk for COPD exacerbations.

However, AGR3 have also been detected in no multiciliated organs such colon, stomach, prostate, and liver (Persson et al., 2005; Brychtova et al., 2014). A recent study showed that AGR3 can promote stemness of colorectal cancer by modulating Wnt/β-catenin signaling (Chi et al., 2020). Wnt signaling functions in branching morphogenesis and airway formation in the developing lung, maintains an epithelial cell phenotype, and forms proper cell-cell junctions (Pongracz & Stockley, 2006). The junctional complexes between neighboring cells consist of TJs and AJs, which also maintain airway epithelial barriers (Nawijn et al., 2011). Previous studies have shown that occludin, ZO-1, and E-cadherin expression in bronchial epithelial cells and lung tissue sections from patients with COPD is lower than that in healthy individuals (Heijink et al., 2014; Nishida et al., 2017; Aghapour et al., 2018). The disruption of airway epithelial barriers can promote translocation of inhaled particles into the subepithelial space, thereby inducing airway inflammation and innate immune cell response (Rezaee and Georas, 2014). However, to our knowledge, the effect of AGR3 on airway epithelial junction proteins in patients with frequent COPD exacerbations has not been studied. Histochemical analysis showed that the expression of occludin, ZO-1, and E-cadherin in airway epithelial cells of the lung tissue from patients with frequent COPD exacerbations was lower than that from patients with infrequent COPD exacerbations. These results suggest that the injured junction complexes in the airway epithelium of patients with frequent COPD exacerbations damage the airway epithelial physical barrier and promote microbial invasion, leading to COPD exacerbation.

Despite evidence of the association between AGR3 and Wnt signaling (Chi et al., 2020), there have been no reports about the effects of AGR3 on airway epithelial cell junction protein expression. Our results showed that AGR3 silencing or overexpression can decrease or increase the expression of occludin, ZO-1, and E-cadherin. Furthermore, AGR3 attenuated CSE-induced downregulation of occludin, ZO-1, and E-cadherin expression. AGR family was first found from X. laevis. In X. laevis, the XAG family of genes appear to be important factors during differentiation of the cement gland (Sive et al., 1989), and human AGR genes had shown to be involved in the epithelial barrier function in inflammatory bowel disease (Zheng et al., 2006). Previous studies showed that AGR2 play a crucial role for maintaining the epithelial phenotype by preventing the expression of E-cadherin (Sommerova et al., 2017), and the E-cadherin-mediated adhesion was shown to specifically promote cell signaling toward formation of other junctions (van Roy and Berx, 2008). AGR3 is a highly related homologue of AGR2, and may be involved in the formation of epithelial junctions through maintaining the expression of E-cadherin. Moreover, AGR3 had shown to promote Wnt/β-catenin signaling (Chi et al., 2020) which play a role to form cell-cell junctions. Although the protective mechanism of AGR3 remains unknown, future studies are warranted to identify the molecular role of AGR3 in cellular junction activity and signaling.

A strict selection criterion for enrollment of eligible patients was implemented in our study. To eliminate individual differences within a group, the patients were grouped according to age, sex, lung function, and tumor type. All lung tissues were tumor free and were obtained from areas away from the tumor. Despite our promising results, our study has several limitations. It was not possible to collect sufficient sputum and BALF samples in patients with stable COPD for AGR3 analysis in future studies. Moreover, comparison between the exacerbation stage and stable stage for frequent COPD exacerbations was not performed due to inability to obtain lung tissue sample during the COPD exacerbation stage.

In our previous study, proteomic analysis showed that AGR3 expression in the lung tissues of patients with frequent COPD exacerbations were low. Herein, we showed that the expression of AGR3 and cell junction proteins (occludin, ZO-1, and E-cadherin) in the lung tissues of patients with frequent COPD exacerbations were decreased. AGR3 plays a role in regulating the expression of junction proteins and can revert CSE-induced downregulation of occludin, ZO-1, and E-cadherin. Thus, AGR3 deficiency can disrupt airway epithelial barriers and enhance translocation of inhaled particles into the subepithelial space to induce airway inflammatory responses, which might increase susceptibility to COPD exacerbation.

## Data Availability

The raw data supporting the conclusion of this article will be made available by the authors, without undue reservation.

## Abbreviations

AGR, anterior gradient; AGR2, anterior gradient 2; AGR3, anterior gradient 3; AJ, adherens junction; COPD, chronic obstructive pulmonary disease; CSE, cigarette smoke extract; PDI, protein disulphide isomerase; TJ, tight junction
